# Case Report: Paraneoplastic psoriasis in thymic carcinoma

**DOI:** 10.3389/fonc.2023.1218517

**Published:** 2023-08-16

**Authors:** Lucas Mix, Manuel Knoll, Max-Felix Häring, Wolfgang Andreas Bethge, Jan C. Schröder, Stephan Forchhammer, Patrick Krumm, Christian M. Schürch, Martin Schaller, Claudia Lengerke

**Affiliations:** ^1^ Department of Internal Medicine II, Hematology, Oncology, Clinical Immunology and Rheumatology, University Hospital Tübingen, Tübingen, Germany; ^2^ Department of Dermatology, University Hospital Tübingen, Tübingen, Germany; ^3^ Department of Radiology, Diagnostic and Interventional Radiology, University Hospital Tübingen, Tübingen, Germany; ^4^ Department of Pathology and Neuropathology, University Hospital and Comprehensive Cancer Center Tübingen, Tübingen, Germany

**Keywords:** thymic carcinoma, psoriasis, paraneoplastic syndrome, thymic epithelial tumors, mediastinal tumor, ADOC

## Abstract

Thymic carcinomas are exceedingly rare and very aggressive malignancies of the anterior mediastinum. While thymomas exhibit a high association with paraneoplastic syndromes, these phenomena are a rarity in thymic carcinomas. In general, acanthotic syndromes such as acroceratosis neoplastica and acanthosis nigricans maligna are commonly observed as paraneoplastic phenomena in patients with carcinomas. In contrast, psoriasis vulgaris, another acanthotic disease, rarely occurs as a paraneoplasia. We report the case of a 36-year-old patient with progressive thymic carcinoma (undifferentiated carcinoma, T3N2M1a) and paraneoplastic psoriasis occurring ten months before the initial diagnosis of the carcinoma. Over the course of the disease, new psoriatic flares heralded relapse or progression of the carcinoma. To our knowledge, this is the first reported case of paraneoplastic psoriasis in thymic carcinoma.

## 1 Introduction

Thymic carcinomas are exceedingly rare tumors of the anterior mediastinum. Thymic epithelial tumors (TETs), comprising thymomas and thymic carcinomas, have an incidence of only about 1,7 per Million in Europe, with thymic carcinomas accounting for only about 10% of these cases (1). The prognosis of patients with thymic carcinoma is usually poor with five-year overall survival rates ranging from 30 to 50% (2). While thymomas are known to be associated with a variety of paraneoplastic syndromes (3), paraneoplastic phenomena in thymic carcinomas are rare. Documented cases of paraneoplastic syndromes in thymic carcinomas include hematologic [e.g. cryoglobulinemia (4), pure red cell aplasia and immune thrombocytopenia (5)], dermatologic [e.g. dermatomyositis (6, 7), skleromyxedema (8), and Leser-Trélat sign (9)] and neurological diseases [e.g. cerebellar degeneration (10) and myasthenia gravis (11)]. Although dermatologic paraneoplastic syndromes, especially dermatomyositis, seem to occur, no cases of paraneoplastic psoriasis have been reported in patients with thymic carcinoma.

## 2 Case description

In February 2023, a 36-year-old patient with progressive, pleurally metastasized thymic carcinoma (undifferentiated carcinoma, T3N2M1a (12, 13)) came to the oncology day clinic of University Hospital Tübingen for the first cycle of chemotherapy with ADOC (cisplatin, doxorubicin, vincristine and cyclophosphamide) (14). Upon physical examination, we noticed erythematous plaques with few white-silvery scaling on both elbows and knees. Furthermore, the patient experienced moderate pruritus. There were no skin abnormalities on the rest of the body and nails and mucous membranes were also not affected. Joint pain was denied and there was no clinical evidence of any form of arthritis. A family history of chronic skin disease was negated.

**Figure 1:**
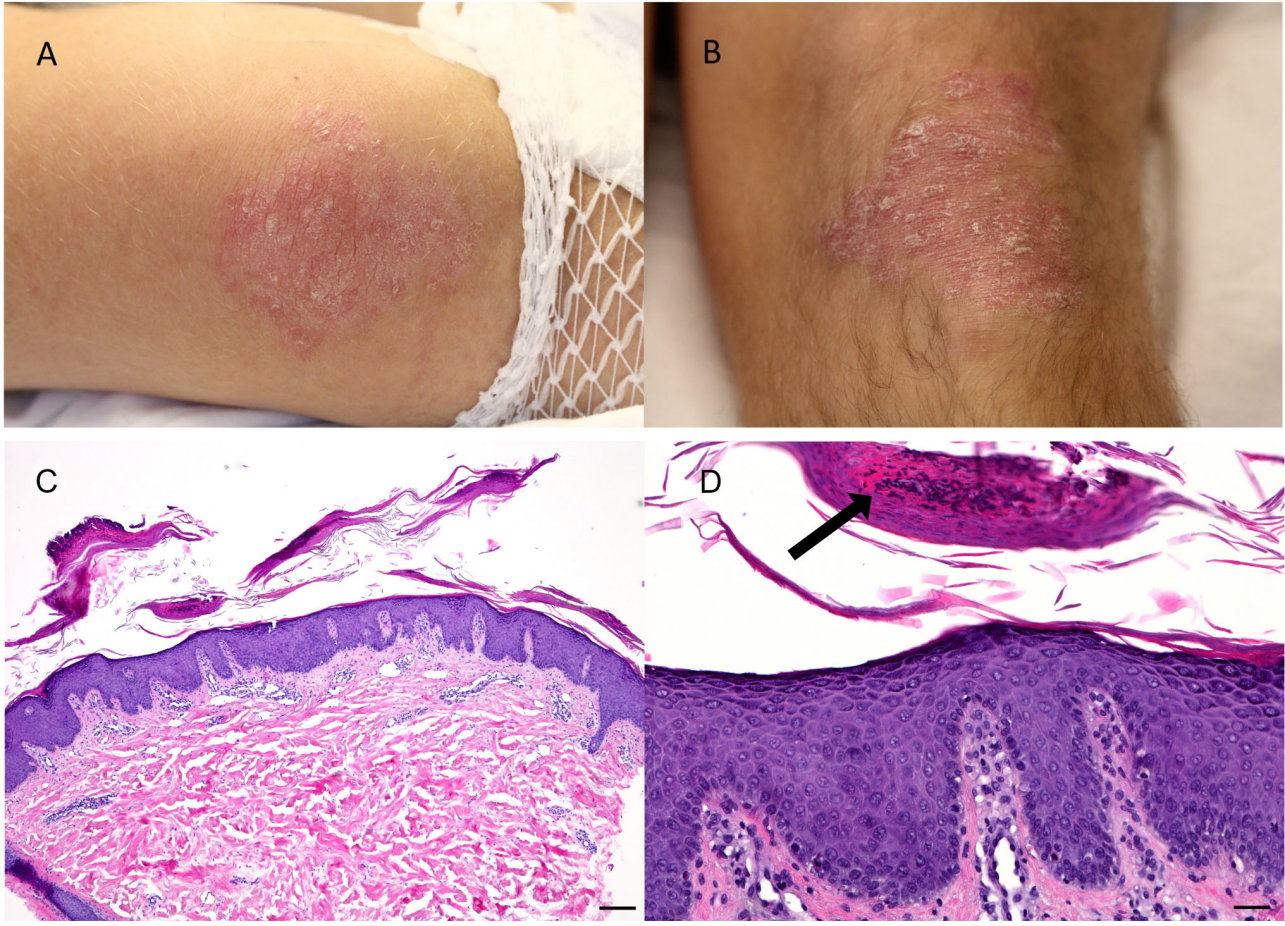
Clinical findings at first presentation and histological findings in a punch biopsy of 4mm: Bilateral erythematous, slightly scaling plaques on elbows **(A)** and knees **(B)** in a 36-year-old patient with thymic carcinoma, without affection of mucous membranes, nail involvement or arthritis. Psoriasiform acanthosis with perivascular lymphocytic infiltration at the upper corium **(C)** (HE, bar 250µm). Magnified depiction of psoriasiform acanthosis with parakeratotic horn and prominent capillary vessels in the papillary dermis, furthermore collection of neutrophils in the stratum corneum (arrow) (HE stain, bar 50µm) **(D)**.

Clinically the skin lesions (**Figure 1**) were consistent with psoriasis vulgaris. This diagnosis was then confirmed by a skin biopsy, revealing psoriasiform acanthosis, parakeratosis, dilated blood vessels in the papillary dermis as well as collections of neutrophils in the stratum corneum (Munro microabscesses). The upper corium showed perivascular lymphocytic infiltrates (**Figure 1**). A complementary PAS stain remained without detection of fungal elements (to exclude the differential diagnosis of tinea corporis).

In September 2019, the patient first noticed an erythematous exanthema of the chest and the upper extremities, which took a self-limiting course over a duration of a few weeks. In November 2019, hyperkeratotic plaques at the elbows and knees were first noticed by the patient. An initial treatment with UVB-311nm phototherapy and topical corticosteroids yielded only unsatisfactory results and was therefore discontinued.

**Figure 2:**
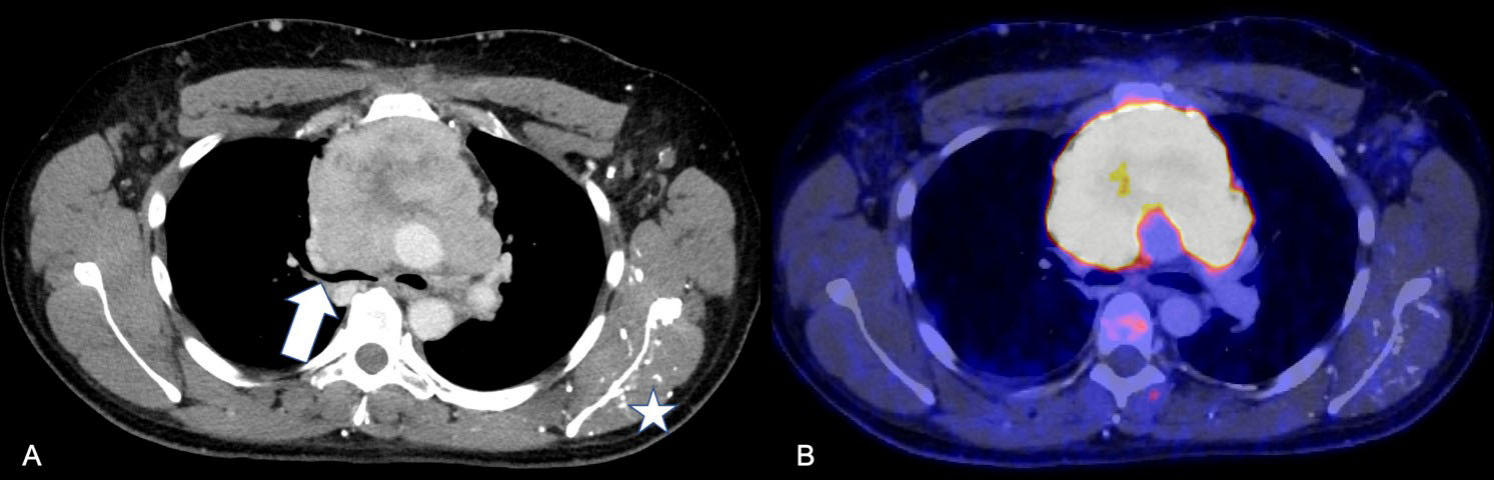
Radiological findings upon initial diagnosis of thymic carcinoma **(A, B)** Baseline hybrid PET/CT: **(A)** Axial contrast-enhanced CT scan demonstrates the large thymic carcinoma mass with sub-total collapse of main bronchi and superior vena cava syndrome (arrow). Influx of i.v. contrast agent applied in left cubital vein via muscular and thoracical veins (star). **(B)** Corresponding 18F FDG-PET fusion overlay indicates high metabolic activity of the tumor (yellow).

**Figure 3:**
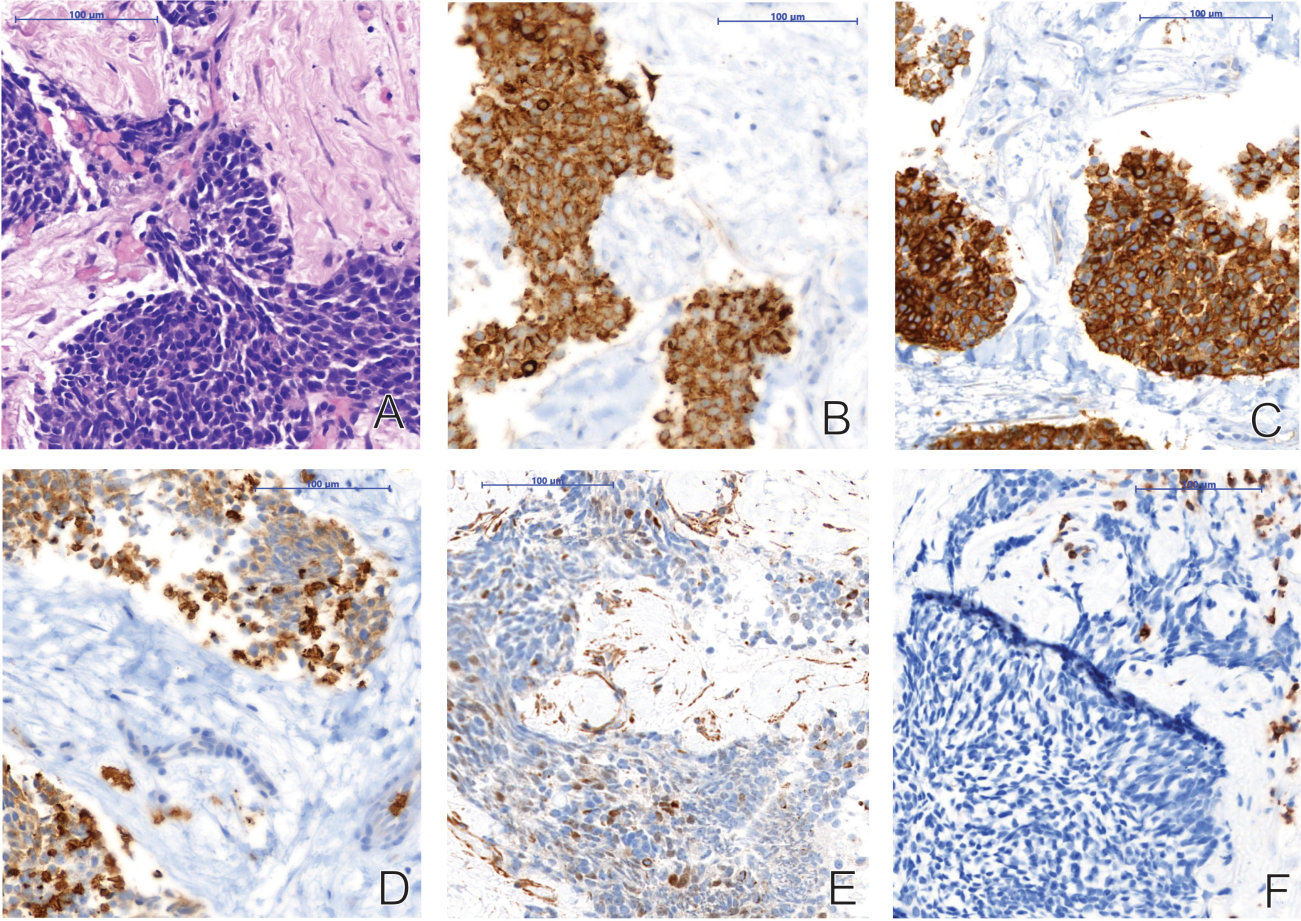
Histological findings of an undifferentiated thymic carcinoma in a CT-guided biopsy of the mediastinal mass upon initial diagnosis: **(A)** HE stain; Tumor islands on a fibrotic background. Small to mid-sized tumor cells with scant eosinophilic cytoplasm and small, ovaloid to spindly, chromatin-dense nuclei. Mitoses are rarely seen. No necrosis. **(B)** Pan-Cytokeratin (AE1/AE3); Diffuse and strong cytoplasmic expression. **(C)** CD117/c-kit; Diffuse and strong cytoplasmic and membranous expression. **(D)** GLUT-1; Diffuse and moderate to strong membranous expression. **(E)** PAX5; Focal, heterogeneous, moderate nuclear expression. **(F)** CD5; Tumor cells negative (T-cells as positive internal control).

In July 2020, the patient presented in our emergency room with progressive dyspnea, thoracic bruising and nocturnal edema of neck and head as a sign of superior vena cava syndrome. Fever, weight-loss or night sweats were negated. A thoracic CT revealed an anterior mediastinal mass with compression of the superior vena cava and the trachea. A PET-CT (**Figure 2**) and a CT-guided biopsy led to the diagnosis of a thymic carcinoma with infiltration of the sternum and the pericardium, as well as pleural and lymphatic metastases [T3N2M1a (12, 13)]. Histological analyses found tumor islands on a fibrotic background. The tumor cells were small to mid-sized, with scant eosinophilic cytoplasm and small, ovaloid to spindly, chromatin-dense nuclei. Mitoses were rarely seen. There was no evidence of necrosis. The HE stain and immunohistochemistry (positive: Pan-Cytokeratin (AE1/AE3), CD117/c-kit, GLUT-1, PAX5 and CD99; negative: CD5, p40, EMA/MUC-1 Synaptophysin, Sall4, PD-L1, Desmin, NUT and Myogenin) were consistent with an undifferentiated carcinoma (**Figure 3**). A targeted next generation sequencing assay (Archer® FusionPlex® Solid Tumor Kit) detected no fusion genes.

After implantation of a tracheal Y-stent, the patient was treated with six cycles of PAC (cisplatin, doxorubicin, vincristine and cyclophosphamide). After the first cycle of treatment, the patient underwent irradiation of the mediastinal tumor mass with 35 Gray and then received cycles two to six of PAC. The following PET-CT found a good overall response to treatment with only one metabolically active retrosternal residue in vicinity to the right atrium.

Interestingly, at this point the psoriatic lesions of the elbow had already vanished completely without specific treatment, with only minimal residual psoriatic lesions at the knees. In January 2021, a lower partial sternotomy was performed to surgically remove the mediastinal residues, along with parts of pericardium and pleura and adjacent adipose tissue. Pathological analyses of the resected tissue found no vital tumor cells. Hence, the patient went into follow up care every three months. In the months following surgery, the residual psoriatic lesions of the knees disappeared completely without any further treatment.

Seven months later, the patient noticed the recurrence of psoriatic lesions on knees and elbows, followed by pain in the right thoracic wall. A thoracic CT scan performed in August 2021 found a nodular mass in the upper mediastinum again and new lesions of the right thoracic wall, as well as in the lower right lobe of the lung. After confirmation of thymic cancer relapse by histopathological analysis of a sonographically guided biopsy, the patient then received a systemic second-line-therapy with paclitaxel/carboplatin for three cycles. After an anaphylactic reaction to paclitaxel, we switched to nab-paclitaxel for cycles four to six. A thoracic CT scan at the end of treatment showed a partial tumor regression. In parallel, the psoriatic lesions had also ameliorated, showing a subtotal regression without any further specific treatment.

During March 2022, the psoriatic skin lesions deteriorated again, with progressive plaques on knees and elbows. A complementary PET-CT scan performed at the end of March 2022 found a progression with new metastases of pleura and thoracic wall. Hence, the patient underwent an off-label therapy with pembrolizumab, which upon progression was switched to a combined therapy with pembrolizumab and lenvatinib. The following CTs in August and November 2022 found a response to therapy with shrinkage of the known metastases. Interestingly, we could not observe any aggravation of the now known psoriasis. On the contrary, a gradual improvement of the psoriasis was noticed with a complete remission of the psoriatic lesions in summer without any specific treatment.

**Figure 4:**
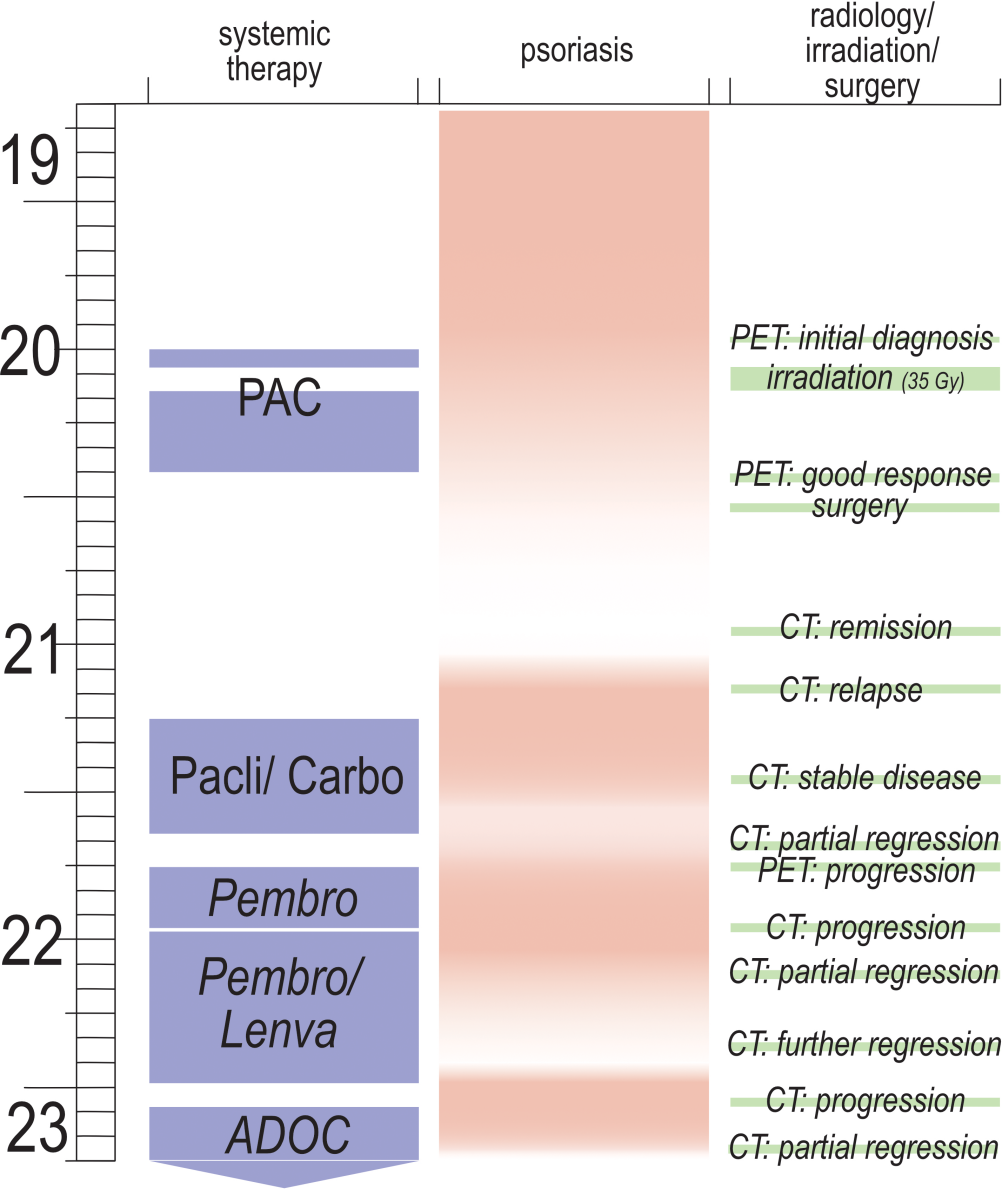
Graphical depiction of treatment of thymic carcinoma, course of psoriasis and course of thymic carcinoma; x-axis= type of systemic therapy, intensity of psoriatic lesions (pink= maximum, white= minimum); y-axis= time in months from September 2019 to March 2023; CT= computed tomography; PET= PET/CT, positron emission tomography and CT; irradiation= three weeks of mediastinal irradiation with 35 Gy; surgery= excision of mediastinal residues, along with parts of pericardium and pleura and adjacent adipose tissue; PAC= cisplatin, doxorubicin, vincristine and cyclophosphamide for six cycles (with a total dosis of 36 mg of dexamethasone per cycle) and irradiation between cycles one and two; Pacli/Carbo= three cycles of carboplatin (AUC6) and paclitaxel (with a cumulative dosis of 32 mg of dexamethasone per cycle), followed by three cycles of carboplatin (AUC6) and nab-paclitaxel (including a total amount of 28 mg of dexamethasone per cycle); Pembro= 4 cycles of monotherapy with pembrolizumab; Pembro/Lenva= 10 cycles of pembrolizumab and lenvatinib; ADOC= three cycles of cisplatin, doxorubicin, vincristine and cyclophosphamide (with a total dosis of 36 mg of dexamethasone per cycle).

In December 2022, the patient noticed the recurrence of psoriasiform lesions on knees and elbows. Four weeks later, after 14 cycles of pembrolizumab (10 of 14 cycles in combination with lenvatinib), the patient presented again with progressive dyspnea. Sonography showed a large pleural effusion on the right side and a thoracic CT scan confirmed the suspected disease progression. Chemotherapy with ADOC (14) (cisplatin, doxorubicin, vincristine and cyclophosphamide) was performed for three cycles until March 2023, when this case report was written. Clinical response was documented with considerable shrinkage of the pleural effusion, cessation of dyspnea and coughing and receding psoriatic plaques on elbows and knees. A thoraxic CT after two cycles of ADOC demonstrated a partial regression of the tumor, especially of the pleural metastases. Given the good response and treatment tolerability, ADOC will be continued until cycle four. **Figure 4** illustrates the chronology of treatment and psoriatic flares.

## 3 Discussion

Paraneoplastic syndromes are common in patients with thymomas (1), but rarely documented for thymic carcinomas (2).

The rarity of paraneoplastic syndromes in thymic carcinomas compared to thymomas can be explained mechanistically. Thymomas retain many histological und functional properties of normal thymic tissue. Thus, they conduct thymopoiesis (15). Abberant thymopoiesis, presumably caused by factors including AIRE-deficiency, reduced MHC-II expression and disrupted organ-architecture, in thymomas leads to the development of autoimmune disorders presenting as paraneoplastic phenomena (16). Thymic carcinomas have lost the ability of thymopoiesis, leading to significantly lower number of immunologic paraneoplastic disorders (16). The pathophysiology of paraneoplastic psoriasis in thymic carcinoma is unclear. No research on the subject has been conducted yet. Hence, further investigations on this topic are needed.

Although it is well known that the onset or exacerbation of psoriasis can be influenced by specific factors such as infections (17–20) or drugs (21), internal malignancies as possible triggers are still disputed (22, 23). In the current literature, only few authors describe an onset of psoriasis in a paraneoplastic context (24, 25). This underlines the rarity and novelty of the case of paraneoplastic psoriasis in thymic carcinoma presented here. Interestingly, other psoriasis-like squamous skin eruptions are well known paraneoplastic comorbidities (26–28).

In this case, our patient presented with typical psoriatic skin lesions, which developed at characteristic locations of psoriasis manifestation. Next to acanthosis, histology revealed an abundant presence of neutrophils in the stratum corneum, which serves as a typical histopathologic hallmark of psoriasis and is not common in other acanthotic paraneoplastic diseases like acanthosis nigricans or Bazex-disease (29–31).

Our patient first noticed psoriasis-like skin eruptions 10 months before the diagnosis of thymic carcinoma was made. In fact, many dermatologic paraneoplastic syndromes tend to present as a prodrome of the underlying malignancy (32). According to the clinical presentation, the severity of psoriasis in our patient can be graded into mild disease (PASI 4,7, DLQI 1). Surprisingly, topical and UV-therapy did not ameliorate the condition, on the contrary the skin manifestation did not change significantly under the treatment with topical glucocorticoids and UVB311nm treatment which is, following the current guidelines in the German speaking area (33), the standard in mild psoriasis treatment. As a consequence of ineffective treatment, our patient discontinued the therapy. Interestingly, the psoriatic lesions vanished after induced remission of the thymic carcinoma under cytostatic therapy without any further specific treatment. Some weeks before the relapse and progression of the thymic carcinoma new flares of psoriasis appeared.

To treat the relapsed thymic carcinoma an Anti- PD1-therapy with pembrolizumab was initiated. Checkpoint-inhibitors have been frequently associated with skin toxicity affecting 16.7% of patients under pembrolizumab treatment (34). Furthermore, those agents are known to induce and exacerbate psoriasis disease (35–38). Typically, psoriasis flares days and months after initiation of PDL1/PD-1-therapy and presents with erythematous plaques and scaling while only few cases of guttata and palmoplantar presentations are reported (37, 39, 40).

Instead, in our patient psoriasis receded under regression of the tumor despite continued exposure to pembrolizumab and flared up again, only weeks prior to the next documented carcinoma progression.

The close correlation between the evolution of psoriatic lesions and thymic carcinoma tumor burden is highly suggestive of a paraneoplastic psoriasis in this case, albeit this cannot be proven. Any form of psoriasis, paraneoplastic or not, could be expected to ameliorate under cytostatic chemotherapy. Furthermore, one should consider that all of the applied chemotherapy regimen contained corticosteroids which have the ability of an initial amelioration of psoriasis. However, systemic glucocorticoid treatment leads to a dramatic deterioration of the disease in the further course according to clinical experience and the literature. Psoriasis flares following systemic glucocorticoid exposure have been repeatedly described in literature and might lead to erythroderma, generalized pustular psoriasis or an exacerbated plaque psoriasis (41–43). Systemic glucocorticoids are therefore no approved drug in the management of psoriasis and are not recommended at the current or earlier versions of the German speaking guidelines (33, 44). With regard to our case our patient did neither show a sudden improvement of his psoriasis after having received high doses of potent glucocorticoids, nor did he present a subsequent disease worsening after systemic glucocorticoid intake. In this case we cannot report either a therapeutic response or a deterioration of the described psoriasis depending on the intake of systemic glucocorticoids. Moreover, different temporal aspects of the disease support the hypothesis of a paraneoplastic genesis. First, initial manifestations of psoriasis presented themselves in close temporal association with the initial diagnosis of thymic carcinoma. Second, in first remission of thymic carcinoma, psoriasis also stayed in remission without any further specific treatment. Third, every relapse or further progression was preceded by a new psoriatic flare, making a mere coincidence unlikely. Furthermore, remission from psoriasis was also induced by a combined therapy of pembrolizumab and lenvatinib. In contrast to cytostatic chemotherapy or application of corticosteroids, this treatment cannot be expected to have a direct positive effect on psoriasis, but rather to aggravate it, as outlined above.

In summary, it can be stated that there was close correlation of disease activity of psoriasis with disease activity of thymic carcinoma. This is contrasted by a much weaker correlation of psoriatic disease activity with application of cytostatic therapy or systemic corticosteroids, as is illustrated by **Figure 4** above. Therefore, we are convinced that, to our knowledge, this is the first reported case of paraneoplastic psoriasis in a patient with thymic carcinoma.

## 4 Patient perspective

When this case report was written, the patient was under treatment with ADOC. The patient tolerated the treatment well, with only little toxicity in the form of moderate nausea and reported a good quality of life under therapy. Therapy greatly reduced burdensome dyspnea and coughing, which the patient had experienced due to the large pleural effusion. The patient stated that psoriasis had little impact on his daily life and quality of life, which is reflected by a DLQI score of 1.

## 5 Informed consent

Informed consent was obtained from the patient. The patient consented to publication of details from his patient history, as well as photos.

## 6 Permission to reuse and copyright

No copyrighted material was used in this case report. PD Dr. med. Patrick Krumm, Senior Physician at University Hospital Tübingen, Department of Radiology, gave permission for the use of PET/CT images. Dr. med. Stephan Forchhammer, Senior Physician at University Hospital Tübingen, Department of Dermatology, gave permission for the use of dermatological images. Prof. Dr. med. Christian Schürch, Senior Physician at University Hospital Tübingen, Department of Pathology and Neuropathology, gave permission for the use of histological images.

## 7 Resource identification initiative

To take part in the Resource Identification Initiative, please use the corresponding catalog number and RRID in your current manuscript. For more information about the project and for steps on how to search for an RRID, please click here.

## 8 Life science identifiers

Life Science Identifiers (LSIDs) for ZOOBANK registered names or nomenclatural acts should be listed in the manuscript before the keywords with the following format:

urn:lsid:<Authority>:<Namespace>:<ObjectID>[:<Version>]

## Data availability statement

The datasets presented in this article are not readily available because of ethical and privacy restrictions. Requests to access the datasets should be directed to the corresponding author.

## Ethics statement

Written informed consent was obtained from the individual(s) for the publication of any potentially identifiable images or data included in this article.

## Author contributions

LM wrote the manuscript, created the figures and took the photos. MK wrote the manuscript and performed the skin biopsy. M-FH critically revised the manuscript. WB critically revised the manuscript. JS critically revised the manuscript. SF histologically analyzed the skin biopsy, provided the corresponding images, and critically revised the manuscript. PK analyzed the radiological findings, provided the corresponding images, and critically revised the manuscript. CS analyzed the histological samples of the carcinoma, provided the corresponding images, and critically revised the manuscript. MS critically revised the manuscript. CL wrote and critically revised the manuscript. All authors contributed to the article and approved the submitted version.
